# Bioactive Polysulfate‐Based Nano‐Assemblies Against Virus Infection

**DOI:** 10.1002/smll.202504384

**Published:** 2025-07-20

**Authors:** Guoxin Ma, Mathias Dimde, Kai Ludwig, Latifa Abidal, Julia M. Adler, Ricardo Martin Vidal, Benedikt B. Kaufer, Jakob Trimpert, Chuanxiong Nie, Rainer Haag

**Affiliations:** ^1^ Institute of Chemistry and Biochemistry Freie Universität Berlin Takustr. 3 14195 Berlin Germany; ^2^ Institute of Chemistry and Biochemistry Research Center of Electron Microscopy Freie Universität Berlin Fabeckstr 36a 14195 Berlin Germany; ^3^ Institute of Virology Freie Universität Berlin Robert von Ostertag‐Str. 7 14163 Berlin Germany; ^4^ Department of Diagnostic Medicine and Pathobiology College of Veterinary Medicine Kansas State University Manhattan KS 66506 USA

**Keywords:** antiviral nanoassembly, multivalent nanosystems, polysulfates, virus inhibition

## Abstract

3D nanosystems equipped with polysulfates as binding sites are effective virus inhibitors due to their ability to dynamically deform while adhering to a virus. Here, a new supramolecular nanosystem assembled from a block copolymer consisting of sulfated linear polyglycerol and polytrimethylene carbonate is presented. It exhibits a unique morphology, 100 nm sized spheres with a distinct brush‐like corona. The negatively charged sulfates are distributed on the outer shell and enable exceptional homogeneity of the particles, thereby enhancing the efficiency of multivalent interactions. Various sulfation levels are tested and demonstrated extremely low half‐maximal inhibition concentration (IC_50_) values in plaque reduction assays tested on herpes simplex virus type‐1 (HSV‐1): 0.43, 0.16, and 0.037 µg mL^−1^ of the 45%, 76% and 100% sulfated assemblies, respectively. Using cryo electron microscopy (cryo‐EM), viruses trapped are observed by multiple layers of the nano‐assemblies. Both 76% and 100% sulfated assemblies show therapeutic potential in the post‐infection model. The inhibitory behavior of the 76% and 100% sulfated assemblies is further confirmed against Omicron infection. This work demonstrates that the presented 3D flexible nano‐assemblies can block the virus entry into the host cells with superior morphology and efficiency, establishing them as a promising candidate for antiviral applications.

## Graphical abstract

Nano‐assemblies with a defined brush‐like corona are developed from sulfated polyglycerol to inhibit virus infection. The sulfate residues on the surface can actively bind the virus and entrap it from entering the host cells. This nanosystem is demonstrated potent inhibition against both HSV‐1 and SARs‐CoV‐2. It also shows therapeutic potential in the post‐infection stage.

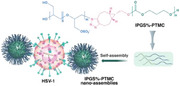

## 1 Introduction

Infectious diseases cause ≈20% of deaths worldwide, with highly pathogenic viruses responsible for about one‐third of these deaths.^[^ ^1^ ^]^ For example, HSV‐1, currently one of the most prevalent viruses in the human population,^[^ ^2^ ^]^ can lead to severe manifestations of diseases, such as corneal blindness,^[^ ^3^ ^]^ HSV‐1 encephalitis, and HSV‐1‐associated neurodegeneration.^[^ ^4^ ^]^ Since it can adopt a latent form during treatment, the HSV‐1 always establishes a life‐long infection and causes recurrence.^[^ ^5^ ^]^ The recent COVID‐19 pandemic has caused incalculable loss of life and economic damage.^[^ ^6^ ^]^ Looking back on the history of human's fight against viral pathogens, we started with hygiene, and took centuries to eliminate human smallpox via vaccination.^[^ ^7^ ^]^ While vaccines have transformed humans’ relationship with viruses, they are limited in quantity and in categories, and they are not equally enforced in all countries.^[^ ^8^ ^]^ Meanwhile, the rapid evolution of viruses is challenging the development of therapeutic approaches.^[^ ^9^, ^10^ ^]^

**Scheme 1:**
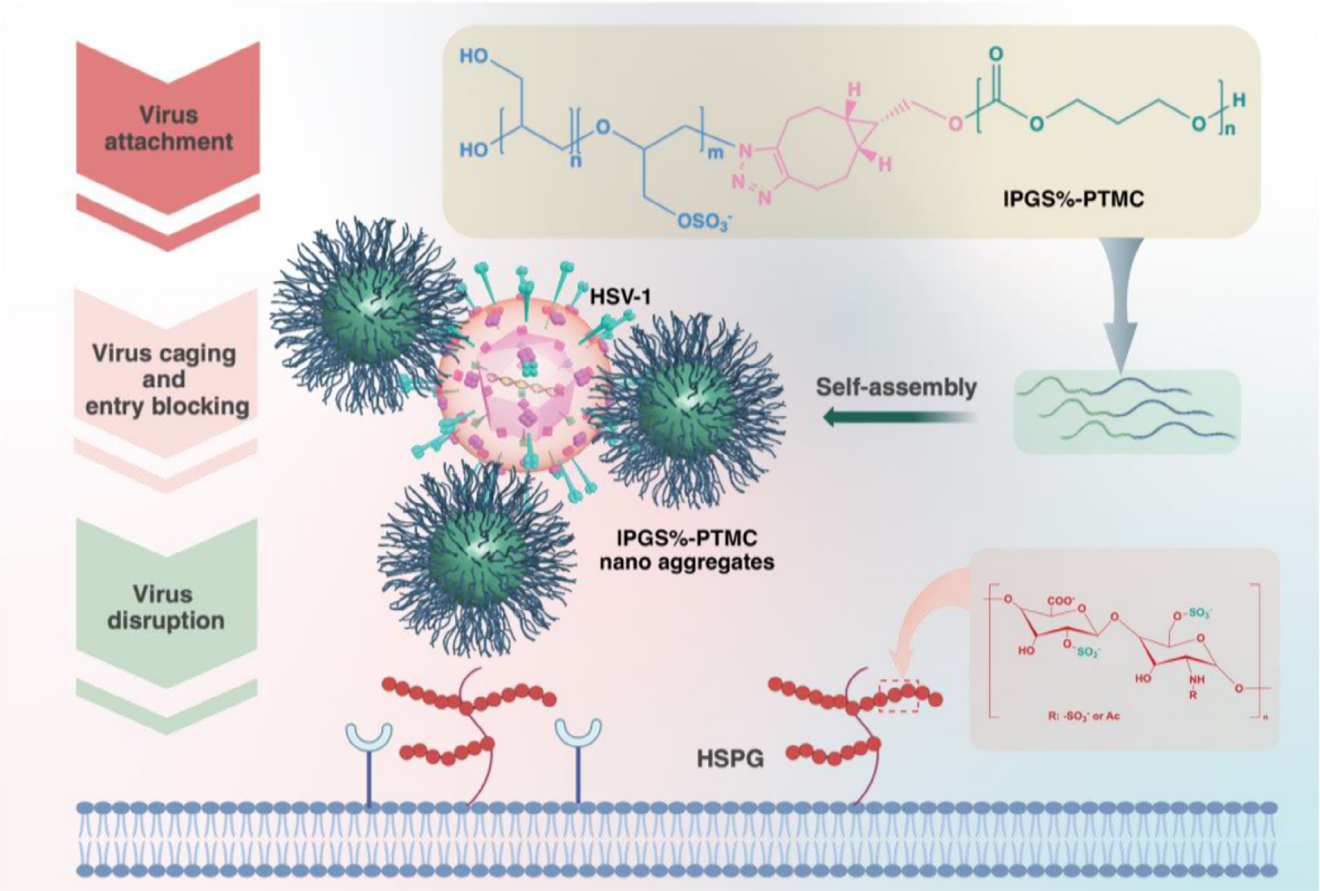
Sulfated nano‐assemblies inhibit HSV‐1 via multivalent interactions.

Research in recent years has focused on understanding the viral infection pathways and developing innovative inhibitors to prevent the infection at an early stage. For instance, various viruses, including HSV‐1, human immunodeficiency viruses, respiratory syncytial virus, and SARS‐CoV‐2, enter the host cells by binding to the heparan sulfate proteoglycans (HSPGs) on the cell surface.^[^ ^11^, ^12^, ^13^, ^14^, ^15^ ^]^ Based on this binding affinity, natural heparin has been used as a binding decoy to inhibit such viruses.^[^ ^16^, ^17^ ^]^ However, the application of natural heparin and its catalogs has been limited by their heterogeneity and by potential risks such as anticoagulant effects.^[^ ^18^, ^19^ ^]^ Moreover, as the binding affinity was found to be dominated by electrostatic interactions, further optimizing the charge density can even strengthen the binding efficiency. Therefore, synthetic heparin mimetics were developed as alternatives to native heparin, and they showed high inhibitory efficiency against a broad antiviral spectrum.^[^ ^20^, ^21^ ^]^ Moreover, these synthetic mimics offer versatility with respect to chain length, morphology, flexibility, and crucially the opportunity for further functionalization, such as grafting them onto other surfaces.^[^ ^22^, ^23^, ^24^ ^]^

In our previous research, we have found that the sulfated linear polyglycerol (lPGS) showed potent inhibition toward both HSV‐1^[^ ^25^ ^]^ and SARS‐CoV‐2.^[^ ^26^ ^]^ Although its linear structure imparts stronger antiviral activity due to its superior flexibility, lPGS, in a size range of 9 nm, can be easily eliminated by renal clearance without executing any inhibitory events. The HSV‐1 typically presents as an enveloped sphere with a size range of 155–240 nm.^[^ ^2^ ^]^ Therefore, we hypothesize that the nano‐assemblies assembled from lPGS at a scale similar to that of the virus can not only adapt to the viral surface and bind the virus with multivalent interactions, but also prolonging the retention time in circulation. Such soft 3D nano‐assemblies were proven to adapt to the viral surface and dynamically trap the virus, eventually causing its local distortion and irreversible deformation.^[^ ^27^, ^28^ ^]^ Various virus‐scale nano‐inhibitors have been demonstrated to have potent antiviral activity, including sulfated nanogels against HSV‐1 infection^[^ ^29^ ^]^ and sialylated nanogels against influenza A virus.^[^ ^30^ ^]^ These inhibitors’ physical attributes are of course, crucial to their effectiveness at virus binding; along with flexibility and proper size, the density of multivalent binding units on the inhibitor is the most crucial factor.^[^ ^31^ ^]^ It is also found that some nanosystems can permanently deactivate a virus through irreversible interactions with the virus’ envelope, endowing their broad‐spectrum antiviral properties. These nanosystems are categorized as virucidal inhibitors. They show great potential to be further formulated into antiviral products when both the desired virucidal activities and minor cell toxicity are attained.^[^ ^32^, ^33^ ^]^

Here, we focus more on self‐assembled nanosystems against virus infection and introduce a new core‐shell structured inhibitor against the HSV‐1 and SARS‐CoV‐2 (**Scheme** **1**). We selected polytrimethylene carbonate (PTMC) with superb biocompatibility and biodegradability as the core to support the sulfated linear polyglycerol (lPGS) to bind viruses. We first synthesized an amphiphilic block copolymer of lPGS and PTMC (lPGS%‐PTMC) by copper‐free click reaction. To correlate the inhibition efficiency with functional binding units, we tuned the number of sulfates on the lPG block while holding the chain length of both blocks constant. We thereby established a series of varying lPGS%‐PTMC sulfation ratios from 23%, 45%, 76% and 100%. Then we performed a simple, fast, and surfactant‐free nanoprecipitation with the lPGS%‐PTMC copolymer, and we surprisingly observed: all the obtained assemblies exhibited a unique morphology: well‐distinguished spheres with brush‐like coronas. We screened the inhibitory efficiency of the assemblies varying the sulfation ratio, by plaque reduction assay, and investigated the binding activity using cryo‐EM. Moreover, we visualized their blocking effect in both pre‐ and post‐infection assays. Finally, we found that the highly sulfated assemblies (76% and 100%) showed the best anti‐viral efficacy against HSV‐1 and the Omicron BA.5 variant of SARS‐CoV‐2.

## 2 Results and Discussion

### 2.1 Design and Fabrication of lPGS%‐PTMC Nano‐Assemblies with Brush‐Like Corona

**Figure 1:**
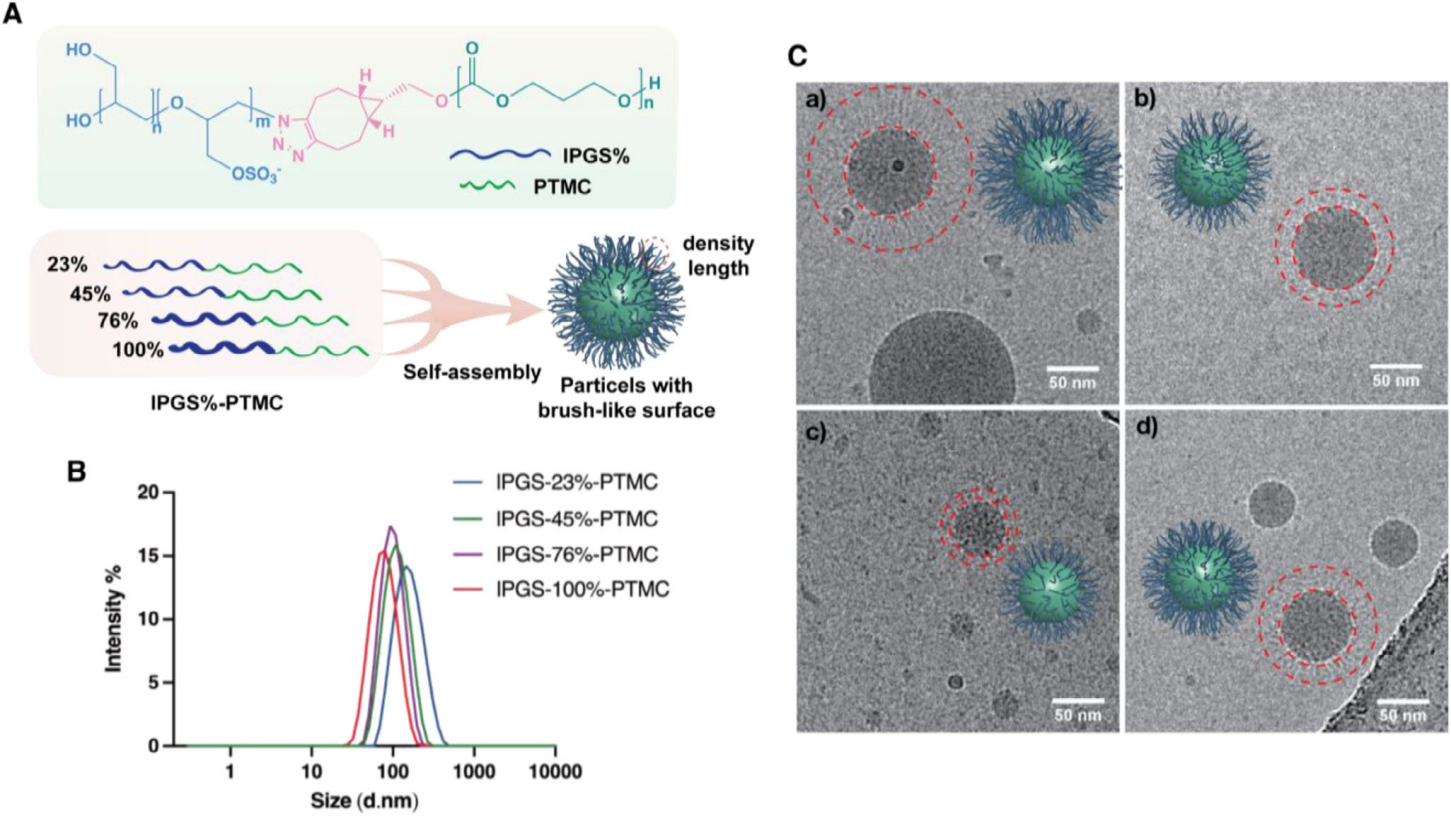
A) Self‐assembly of lPGS%‐PTMC into aggregates with brush‐like corona. B) Size distribution of lPGS%‐PTMC measured by DLS. C) Cryo‐EM images of lPGS%‐PTMC nano‐assemblies: a) lPGS‐23%‐PTMC, b) lPGS‐45%‐PTMC, c) lPGS‐76%‐PTMC, d) lPGS‐100%‐PTMC.

The azide functionalized linear polyglycerol (N_3_‐lPG) was synthesized as reported before.^[^ ^34^ ^]^ The molecular weight of lPG was 8.5 kDa (Figure S1, Supporting Information). Then we sulfated the N_3_‐lPG at varying sulfation ratios at 23%, 45%, 76%, and 100%; we followed our previous protocol and calculated the sulfation ratios by the integration area of ^1^H NMR spectrum (Figure S2, Supporting Information). We synthesized the BCN functionalized polytrimethylene carbonate (BCN‐PTMC) similarly to our previous study,^[^ ^34^ ^]^ and the obtained BCN‐PTMC had a molecular weight of 9.5 kDa (Figures S3, S4, Supporting Information). We then obtained the copolymer of lPGS%‐PTMC by click reaction between N_3_‐lPGS and BCN‐PTMC (Figure S5, Supporting Information). We characterized this copolymer by FTIR, finding that its absorbance of azide at 2100 cm^−1^ disappeared after click; this result demonstrates the completed linkage between lPGS and PTMC (Figure S6, Supporting Information). Afterward, we formulated the nano‐assemblies with the amphiphilic copolymer lPGS%‐PTMC via nanoprecipitation. We characterized the size of the formulated particles by dynamic light scattering (DLS). As shown in **Figure** **1**A, the size of particles decreased as the sulfation ratio increased (Figure 1B). All of the fabricated particles exhibited relatively narrow size distributions, indicating their homogeneity (**Table** **1**). We then studied the morphology of the particles by cryo‐EM. Strikingly, we found that all of the nano‐assemblies were spheres, with individual lPGS chains protruding from the solid PTMC core to form a brush‐like corona whose bristle aggregates varied in density and length depending on the sulfation ratio of the nanoaggregates (Figure 1C). For the lPGS‐23%‐PTMC assemblies, the diameter of the hydrophobic core was similar to the length of bristles on the surface due to the symmetric distribution of the hydrophobic and hydrophilic chains (Figure 1B). The lPGS‐45%‐PTMC assemblies showed a slight decrease in volume of their hydrophobic cores but greatly shortened brush height on their surfaces. Since the molecular weight of the PTMC block is identical among all the assemblies, the relative hydrophobicity of the PTMC block weakened as the degree of sulfation increased. The strong hydrophilic interactions within the lPGS blocks and between the lPGS chains and water molecules caused the compression of the hydrophobic core and led to overall smaller particles. In addition to the sulfation‐dependent size differences, we observed that the length of the brush structure on the surface first decreased with higher sulfation ratio of the lPG, then increased again when the sulfation ratio was raised up from 76% to 100%. We believe that the nano‐assemblies’ inhibition activity of relies not only on the length of the brush structure but also on the charge density on the brushes. However, the density of the brush‐like structure is rather difficult to define from the cryo‐EM images. We investigated the zeta potential of the nano‐assemblies with four different sulfation ratios. As we expected, all of these assemblies showed negative zeta potential, with the value decreasing at higher sulfation ratios. The lPGS‐100%‐PTMC assemblies exhibited the highest number of charges, indicating the especially high exposure of sulfates on their surfaces. To evaluate the inhibitory efficiency of the lPGS%‐PTMC assemblies and to confirm the critical factors for their antiviral activity, we continued the bioassay with the HSV‐1.

**Table 1:** Size and zeta potential of nano‐assemblies (data is expressed as mean ± SD, *n* = 3).

lPGS%‐PTMC	Size [nm]	PDI	Zeta Potential [mV]
23%	150.4	0.19	−13.7 ± 1.6
45%	115.6	0.09	−25.5 ± 1.9
76%	92.9	0.08	−33.6 ± 0.8
100%	78.8	0.12	−44.2 ± 1.1

### 2.2 Inhibitory Activity of lPGS%‐PTMC Nano‐Assemblies Against HSV‐1

**Figure 2:**
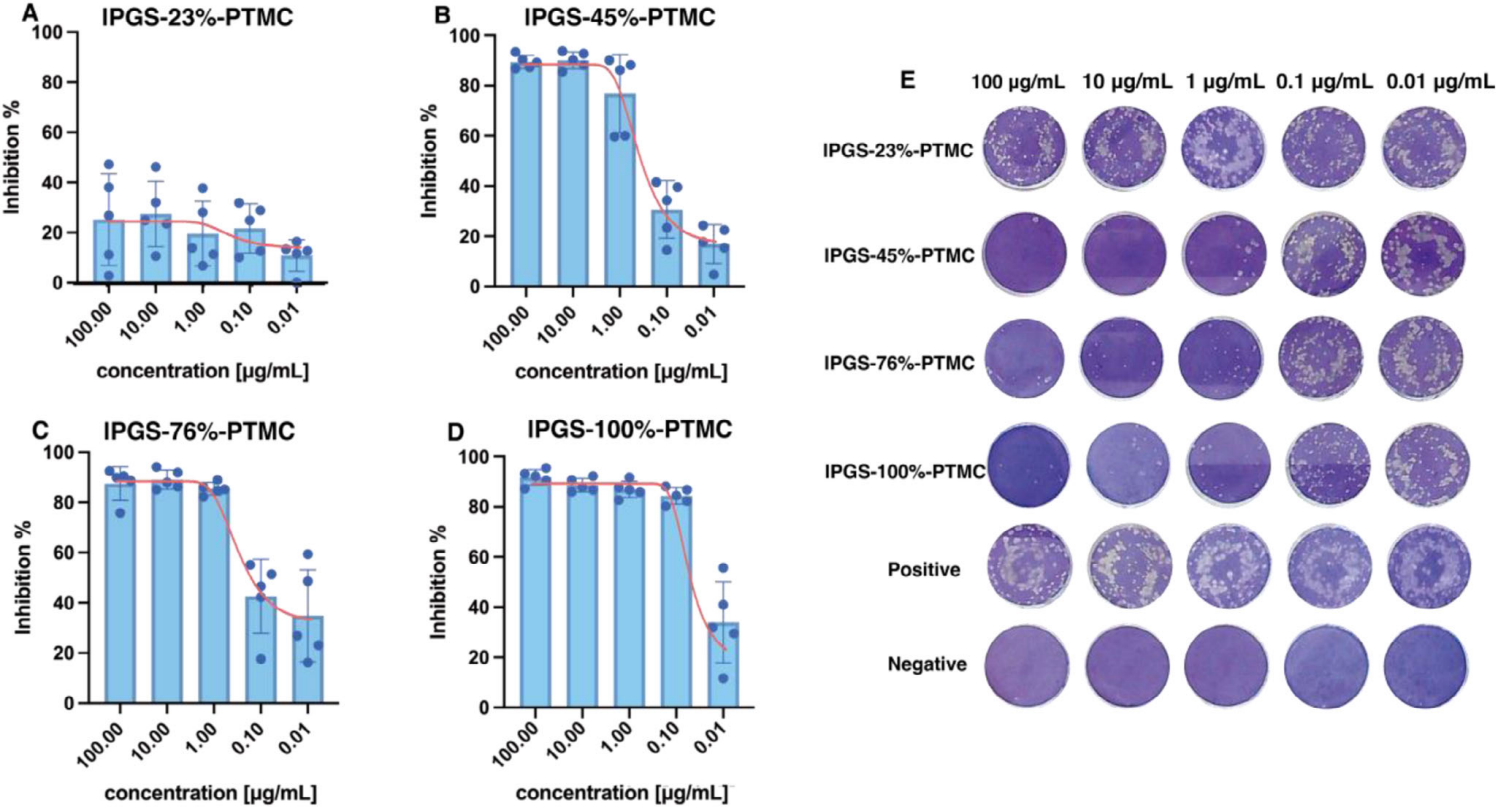
Concentration‐dependent inhibition efficacy against HSV‐1 of lPGS‐23%‐PTMC A), lPGS‐45%‐PTMC B), lPGS‐76%‐PTMC C), and lPGS‐100%‐PTMC D) via plaque reduction assay (mean ± SD, *n* = 5). The Inhibition curve is individually indicated in red in each graph, and it's analyzed by log(inhibitor) versus response – variable slope model embedded in Prism. E) Images of crystal violet‐stained Vero E6 cells infected by HSV‐1.

Based on our previous study, the antiviral activity of lPGS is dependent on its quantity of sulfates.^[^ ^26^ ^]^ To confirm our hypothesis, we evaluated the inhibition efficiency of the lPGS%‐PTMC assemblies with the plaque reduction assay. The HSV‐1 was individually pre‐treated with the lPGS%‐PTMC assemblies of varying sulfation degrees, then incubated with Vero E6 cells. We quantitatively analyzed the number of plaques of each group, and the dose‐dependent inhibition efficiency of each sample is shown in **Figure** **2**. We found that lPGS‐23%‐PTMC assemblies displayed no inhibition toward HSV‐1, even at the high concentration of 100 µg/mL. When the assemblies’ sulfation ratio was increased to 45%, we observed potent viral inhibition, with a IC_50_ of ≈0.43 µg mL^−1^. This result endorses 45% as the critical sulfation degree for the assemblies to display viral inhibition. Moreover, the IC_50_ values of lPGS‐76%‐PTMC and lPGS‐100%‐PTMC assemblies were measured as 0.16 and 0.037 µg mL^−1^, respectively (Figure S7, Supporting Information). In contrast, the commercial drugs to treat HSV‐1 infection, valaciclovir, acyclovir, and famciclovir exhibit IC50 of 0.29, 0.19, and 0.86 µg mL^−1^, respectively.^[^ ^35^, ^36^, ^37^ ^]^ Our 100% sulfated nanoassemblies showed a nearly tenfold decrease in IC50 than these commonly used antiviral therapeutics. These results make clear that the aggregates’ inhibition efficiency can be enhanced by increasing their degree of sulfation. Since an increased degree of sulfation correlates with increased surface charge density on the nano‐assemblies’ brush‐like corona, these results indicate that the sulfation ratio or the quantity of exposed sulfates is the crucial factor in the assemblies’ antiviral performance. We have verified that the inhibitory efficiency is proportional to the sulfation degree for linear polyglycerol chains.^[^ ^25^ ^]^ However, for the sulfated lPG‐based assemblies, the non‐linear correlation between IC_50_ value and the sulfation ratio of each nano‐assemblies suggests that the inhibition efficiency is more likely dependent on multiple factors, such as size, brush length, and brush flexibility, and so on. The size of nanoassemblies decreased when the sulfation level increased. As we know, smaller particles tend to aggregate on the surface of binders and therefore, are more active than the larger ones.^[^ ^38^ ^]^ In our case, the 100% sulfated nano‐assemblies showed the smallest size, and this can therefore increase the proportion of particles binding to one virus. Moreover, we have seen in our previous study that the soft and flexible nanogels are 400‐times more efficient than the rigid nanogels in inhibiting influenza.^[^ ^30^ ^]^ We, therefore, suspect that the binding efficiency of the brush‐like surface derives not only from the number of sulfate residues but also from the assemblies’ hydrophilicity or flexibility, both of which are dependent on the degree of sulfation.

**Figure 3:**
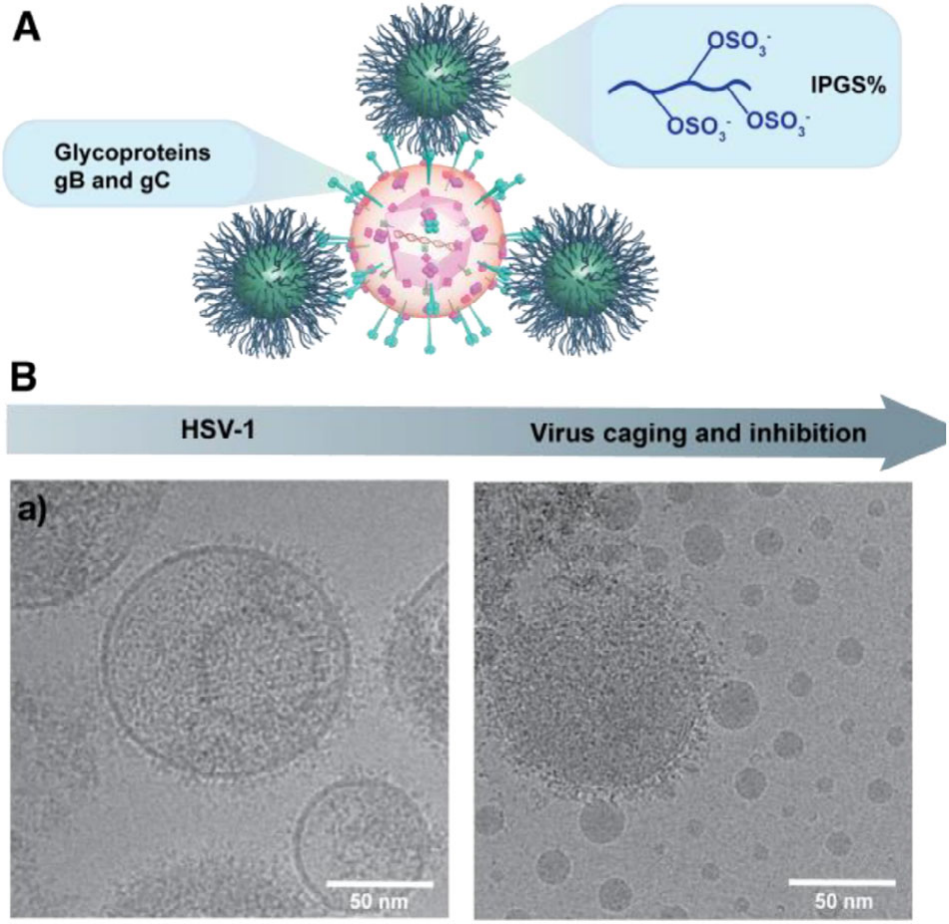
A) Dynamic inhibiting behavior of lPGS‐100%‐PTMC nano‐assemblies. B) Cryo‐EM images of lPGS‐100%‐PTMC nano‐assemblies with HSV‐1: a) Native HSV‐1 with glycoproteins on the envelope. b) Multivalent binding and trapping of HSV‐1 by lPGS‐100%‐PTMC nano‐assemblies (scale bar: 50 nm).

We furthermore inspected the interactions between the nano‐assemblies and the HSV‐1 virus by cryo‐EM. As illustrated in **Figure** **3**A, the sulfates on the lPG chain are homogeneously distributed in the water phase, achieve multivalent binding with the glycoproteins gB and gC, which regulate fusion of HSV‐1 by attachment to the HSPG receptors on host cells.^[^ ^14^ ^]^ In Figure 3B, we can see the distinct morphological units of untreated HSV‐1, including the DNA‐containing capsid and the lipid‐bilayer envelope. After incubation with lPGS‐100%‐PTMC assemblies, we observed that the virus was trapped by the aggregates, homogeneously distributed all around it, and this active capture can powerfully inhibit viral escape. This finding suggests that our nano‐assemblies can trap viruses via dynamic electrostatic interactions. Moreover, the sulfated nanoassemblies exhibited weak virucidal effects (Figure S8, Supporting Information), and this clearly indicates that the inhibitory events were attributed to shielding the virus‐host interactions instead of virucidal actions. Taking together the results of virus inhibition, we hypothesize that this interaction drives the nano‐assemblies to inhibit the virus from entering the host cells. Figure 3B also shows nano‐assemblies adhered to the partially disrupted envelope. We believe that the nano‐assemblies’ electrostatic inter‐repulsion not only leads to their homogeneous distribution but may also strengthen the potency of their virus inhibition.

### 2.3 Fluorescent Visualization of lPGS%‐PTMC Nano‐Assemblies Inhibiting Virus

**Figure 4:**
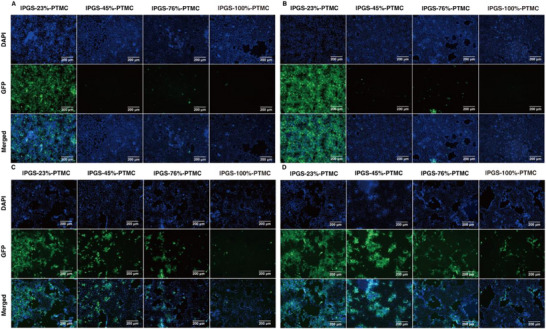
Representative fluorescent images of Vero E6 cells infected by pre‐inhibited HSV‐1 with nano‐assemblies lPGS‐23%‐PTMC, lPGS‐45%‐PTMC, lPGS‐76%‐PTMC, and lPGS‐100%‐PTMC at a concentration of 100 µg mL^−1^ A) and at a concentration of 10 µg mL^−1^ B). Representative fluorescent images of Vero E6 cells infected by HSV‐1 and post‐incubated with nano‐assemblies lPGS‐23%‐PTMC, lPGS‐45%‐PTMC, lPGS‐76%‐PTMC, and lPGS‐100%‐PTMC at a concentration of 100 µg mL^−1^ C) and at a concentration of 10 µg mL^−1^ D).

To further prove the inhibitory activities of lPGS%‐PTMC nano‐assemblies against HSV‐1, we performed infection assays in the presence of the nano‐assemblies. To correlate the inhibitory efficiency with concentration, we also acquired images of lPGS%‐PTMC nano‐assemblies at different concentrations: 100 and 10 µg mL^−1^. Compared with the control group (Figure S9, Supporting Information), the lPGS‐23%‐PTMC nano‐assemblies showed no inhibition toward HSV‐1, and most cells were infected. By contrast, the other groups, with sulfation degrees ranging from 45% to 100%, showed potent inhibition of HSV‐1. With only a few cells infected by the end of co‐incubation (**Figure** **4**A,B). This result confirms again that the effective inhibition demands a critical number of sulfate residues; in this case, the required value is ≈60 residues per polymer chain. Furthermore, we carried out a post‐infection assay to evaluate the therapeutic potential of the nano‐assemblies. We used fluorescence microscopy to acquire images of Vero E6 cells pre‐infected by HSV‐1, and then incubated with the various nano‐assemblies at different concentrations of 100 and 10 µg mL^−1^. In the 100 µg mL^−1^ group (Figure 4C), the 23% sulfated nano‐assemblies showed no inhibition in the post‐infection stage; the 45% sulfated nano‐assemblies showed some inhibition; and the percentage of infected cells dropped when we increased the sulfation ratio from 45% to 100%. Strikingly, the 100% sulfated nano‐assemblies showed nearly complete viral inhibition, even after the first infection cycle. After ten‐fold dilution of the nano‐assemblies, viral transmission was enhanced. The 23% and 45% sulfated nano‐assemblies no longer displayed any antiviral events, and the 76% sulfated nano‐assemblies showed only slight inhibition as compared to the control. However, the 100% sulfated group showed only slightly weakened inhibition after tenfold dilution.

We also evaluated the biosafety of the nano‐assemblies by CCK8 assay (Figure S10, Supporting Information). All of the nano‐assemblies across varying sulfation ratios displayed superb biocompatibility even at 1000 µg mL^−1^, a value which is ≈2300‐fold, 6250‐fold, and 27 000‐fold of the IC_50_ of the 45%, 76% and 100% sulfated nano‐assemblies, respectively. All told, the fully sulfated nano‐assemblies showed promising therapeutic efficacy in vitro, and we can adapt the nano‐assemblies to various scenarios by tuning their sulfation ratios and working concentrations.

### 2.4 Inhibition of Omicron BA.5 with lPGS%‐PTMC Nano‐Assemblies

**Figure 5:**
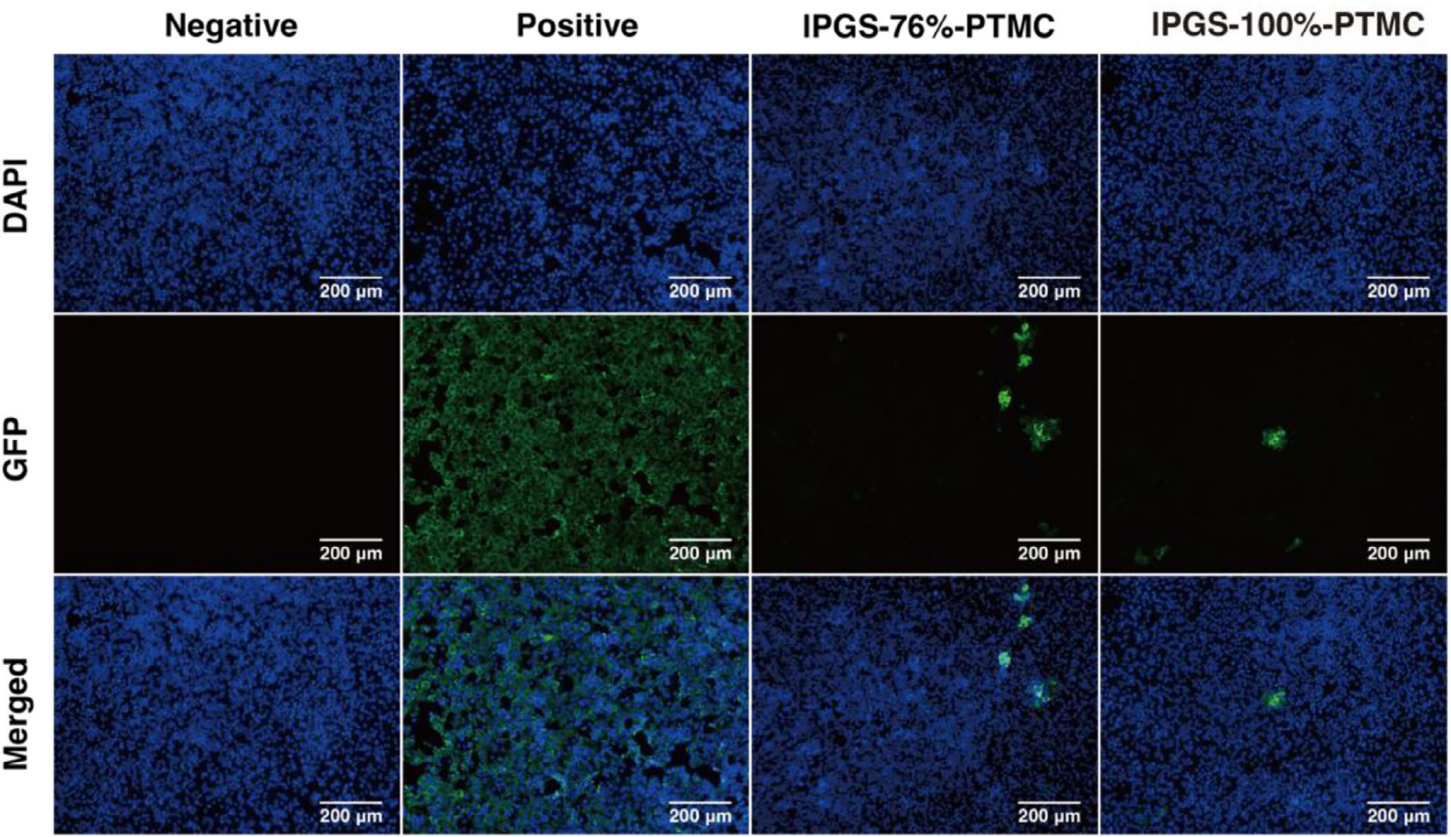
Representative fluorescent images of Vero E6 cells infected by omicron BA.5 pre‐inhibited by nano‐assemblies lPGS‐76%‐PTMC and lPGS‐98%‐PTMC at a concentration of 100 µg mL^−1^.

Finally, our previous work demonstrated that the sulfated lPG shows strong binding affinity toward the highly positively charged receptor binding domain of the SARS‐CoV‐2 spike protein.^[^ ^26^ ^]^ In the present work, after establishing that the lPGS%‐PTMC nano‐assemblies are effective inhibitors against HSV‐1, we further evaluated their inhibitory activity against SARS‐CoV‐2. We selected the nano‐assemblies with relatively high sulfation rates: 76% and 100%. Using these compounds, we performed a pre‐infection assay on the variant of SARS‐CoV‐2, Omicron BA.5, and imaged the infected Vero E6 cells with fluorescent microscopy. As shown in **Figure** **5**, nearly complete infection was observed for the cellular monolayer in the non‐treated positive control group. In contrast, the virus incubated with nano‐assemblies failed to infect the majority of cells, resulting in only a few infectious foci in an otherwise undisturbed cellular monolayer. Our assays also suggested a slightly higher antiviral efficacy for the 100% sulfated nano‐assemblies compared to the 76% sulfated group. We have therefore confirmed that the sulfated lPG nano‐assemblies are effective inhibitors of Omicron BA.5 and showcase the potential of our approach to serve as an effective antiviral strategy against highly distinct groups of enveloped viruses.

## 3 Conclusion

Sulfated polymers can inhibit HSV‐1 and SARS‐CoV‐2 by electrostatic interactions. In recent years, we have devoted our attention to extending the categories of inhibitors from 1D to 2D‐ and 3D assemblies, such as nano‐sheets, nanofibers. In this work, we developed new supramolecular nano‐assemblies. Briefly, we synthesized an amphiphilic block copolymer consisting of sulfated linear polyglycerol with a gradient sulfation ratio and polytrimethylene carbonate. We then employed the copolymer to construct the 3D nano‐assemblies, all of which displayed a unique morphology: a spherical hydrophobic core with a hydrophilic brush‐like surface. The diameter of the hydrophobic core and the length of the bristles varied with the sulfation degree of the lPG chain. The exposed sulfates on the brush‐like surface are crucial for virus binding, and we observed under cryo‐EM that the 100% sulfated nano‐assemblies attached multivalently to the virus. In the bioassays, we established that the assemblies exhibit potent inhibition toward HSV‐1, as long as the sulfation ratio is above 45%; we also found that an increased sulfation ratio improves the assemblies’ inhibitory efficiency. Moreover, we found that the lPGS‐100%‐PTMC nano‐assemblies can significantly reduce the viral transmission in the post‐infection phase, demonstrating the therapeutic potential of these sulfated aggregates. Finally, we evaluated lPGS‐76%‐PTMC and lPGS‐100%‐PTMC nano‐assemblies’ ability to inhibit the Omicron BA.5 variant of SARS‐CoV‐2. Surprisingly, both groups efficiently blocked the infection. We conclude that the lPGS%‐PTMC nano‐assemblies presented in this work can multivalently bind to viruses and efficiently prevent them from entering host cells. Moreover, the 3D nano‐assembly strategy presented in this study can be employed to develop inhibitors against other virus strains, for example, sialic acid functionalized nano‐assemblies against influenza infection.

## Conflict of Interest

The authors declare no conflict of interest.

## Supporting information



Supporting Information

## Data Availability

The data that support the findings of this study are available in the supplementary material of this article.
