# Supplementary material for: Bioactive Polysulfate‐Based Nano‐Assemblies Against Virus Infection
Source: Small. 2025 Jul 20;21(36):e04384. doi: 10.1002/smll.202504384 (PMC12423918; doi:10.1002/smll.202504384)
Supplement: Supplementary file 1 — Supporting Information [file SMLL-21-e04384-s001.docx]

**Supporting Information**

**Bioactive Polysulfate-Based Nano-assemblies against Virus Infection**

Guoxin Ma^a^, Mathias Dimde^b^, Kai Ludwig^b^, Latifa Abidal^a^, Julia M. Adler^c^, Ricardo Martin Vidal^c^, Benedikt B. Kaufer^c^, Jakob Trimpert ^d^, Chuanxiong Nie^a, *^, Rainer Haag^a,^ ^*^

[a] Guoxin Ma, Latifa Abidal, Dr. Chuanxiong Nie, Prof. Dr. Rainer Haag
Institute of Chemistry and Biochemistry

Freie Universität Berlin

Takustr. 3, 14195 Berlin, Germany
E-mail: [chuanxnie@zedat.fu-berlin.de](mailto:chuanxnie@zedat.fu-berlin.de), [haag@zedat.fu-berlin.de](mailto:haag@zedat.fu-berlin.de)

[b] Dr. Kai Ludwig, Dr. Mathias Dimde

Institute of Chemistry and Biochemistry

Research Center of Electron Microscopy

Freie Universität Berlin,

Fabeckstr 36a, 14195 Berlin, Germany

[c] Julia M. Adler, Ricardo Martin Vidal, Prof. Dr. Benedikt B. Kaufer

Institute of Virology

Freie Universität Berlin

Robert von Ostertag-Str. 7, 14163, Berlin, Germany

[d] Dr. Jakob Trimpert

Department of Diagnostic Medicine and Pathobiology

College of Veterinary Medicine

Kansas State University

Manhattan, KS, USA

**Experimental section**

**Material**

Sodium azide, 2, 2-bis(bromomethyl) 1, 3-propanediol, triazabicyclo[4.4.0]dec-5-ene (TBD), Anhydrous dichloromethane (≥ 99.8%, contains 40-150 ppm amylene as stabilizer), anhydrous tetrahydrofuran (≥ 99.9%, inhibitor-free), Anhydrous dimethylformamide (99.8%), methylcellulose were ordered from Sigma, USA. Poly-(Ethoxyethyl Glycidyl Ether) (PEEGE) of 20 kDa was synthesized by Daniel Kutifa (Freie Universität Berlin) as reported before.^[1]^ Trimethylene carbonate (TMC) was provided by the Group of Prof. Zhiyuan Zhong, Soochow University.^[2]^ Dulbecco’s modified eagle medium, RPMI medium 1640, 0.05% trypsin-EDTA (1X), DPBS buffer and feta bovine serum were purchased from Gibco, USA. Cell counting kit-8 were acquired from Thermo Fischer. Vero E6 cells (CRL-1586) were acquired from ATCC, USA. The GFP tagged HSV-1 was kindly provided by Dr. Yasushi Kawaguchi, University of Tokyo, Japan. The SARS-CoV-2 nucleocapsid (N) Protein Antibody was ordered from Invitrogen, Germany. The Hoechst and goat anti-mouse IgG (Alexa 488 conjugated) were purchased from Thermofisher, Germany.

**Instrument**

The polymer structures were determined by ^1^H NMR conducted on spectrometer ECX 400 (JEOL, USA) at 400 MHz. The chemical shifts were calibrated against residue solvent signals. The molecular weight and polydispersity index were measured by gel permeation chromatograph (GPC) LC-2030 produced by Shimadzu, Japan. The characterization of polymer was performed by THF or milli-Q water as the eluent at a flow rate of 1 mL/min. The calibration for columns was carried out by series of Polystyrene and sample concentration of 5 mg/mL was required. The diameter, size distribution of nanogels were determined by dynamic light scattering (DLS, Zetasizer ultra, Malvern Panalytical, UK) equipped with a 632.8 nm He-Ne laser beam alongside non-invasive back scattering technology. Specimen preparation for cryogenic transmission electron microscopy (cryo-TEM) was performed by plunge-freezing using an FEI Vitrobot Mark IV (Thermo Fisher Scientific Inc., Waltham, Massachusetts, USA). Absorbance of cells treated with CCK-8 assay were measured by microplate reader, Tecan Spark, Switzerland. Fluorescence microscope, Zeiss Axis observer Z1 colorcam, Germany was used for visualization of HSV-1 and Omicron BA.5 infected Vero E6 cells.

**Synthesis of N_3_-lPGS%**

Sulfated linear polyglycerol (lPG) was synthesized as the protocol reported before.^[3]^ The completely dried lPG (1000 mg, 0.1 mmol,1 eq) was added to a pre-dried schlenk flask under ambient atmosphere. Then anhydrous dimethylformamide (20 mL) was added and the mixture was heated up to 80 ºC. After lPG dissolved, sodium azide (32.5 mg, 0.5 mmol, 5 eq) was carefully added, and the reaction runs for 3 days at 80 ºC under vigorous stirring. The precipitation was removed by filtration and the crude product was dialyzed in MeOH for 2 days with intensive medium exchange. The sulfation of N_3_-lPG to different degrees is described as followed. To pre-dried Schlenk flasks, anhydrous N_3_-lPG (200 mg, 0.02 mmol, 1 eq) is added individually and dissolved in dry dimethylformamide (5 mL). The mixture is heated up to 60 ºC and then respective amounts of SO_3_/Triethylamine complex (25%, 50%, 75%, 100%) are added under inert flow. The reaction proceeded at 60 ºC for 24 h. Afterwards, the pH of the mixture was adjusted to 9 by dropwise addition of 1 M NaOH solution. Thereafter, the mixture was dialyzed (MWCO = 2 kDa) against saturated NaCl solution for 2 days and D.I water for 2 days. Then the product is collected after lyophilization. The degree of sulfation is calculated according to ^1^H NMR spectrum.

**Synthesis of BCN-PTMC**

BCN functionalized polytrimethylene carbonate (BCN-PTMC) was obtained by ring opening polymerization of trimethylne carbonate (TMC) initiated by (1R,8S,9s)-Bicyclo[6.1.0]non-4-in-9-yl-methanol (BCN-OH). TMC was synthesized similarly according to the previously introduced protocol.^[2]^ Typically, stock solution of organo-catalyst TBD (0.1 mM, 1 mL) in dry DCM and BCN-OH (0.1 mM, 2 mL) in dry DMSO were prepared under inert flow. With respective ratio, solution of TBD (0.2 mL) and BCN-OH (1 mL) were added to reactor with TMC (728.91 mg, 7.14 mmol, 1.05 eq) dissolved in anhydrous DMSO (10 mL) under stirring. The reaction proceeded at 50 ºC for 24 h. The polymerization was quenched after 90 min by methanol (0.1 mL). Then the crude product was precipitated in cold hexane (100 mL), centrifuged and repeated for 3 times. Afterwards, the precipitates were filtered and wash by cold hexane (3 × 10 mL). White solids of BCN-PTMC were finally obtained via vacuum drying.

**Synthesis of lPGS%-PTMC and fabrication of nano-assemblies**

The serial block copolymer of lPGS%-PTMC varying sulfation rate is obtained via SPAAC click reaction. Briefly, azide-lPGS% (1 eq) and BCN-PTMC (1.1 eq) were separately dissolved in DMSO. Then the solution of BCN-PTMC was injected to the solution of azide-lPGS% and the reaction proceeded under vigorous stirring for 24 hours. Then the mixture was dialyzed against water (MWCO = 2 kDa) with intensive exchange and the crude product was obtained after lyophilization. Next, the crude product was precipitated in DCM for three times and collected as white solids after vacuum drying.

**Characterization of lPGS%-PTMC nano-assemblies**

The nano-assemblies are fabricated via nanoprecipitation. Typically, to 900 µL of phosphate buffer (PB, pH=7.5, 10mM), 100 µL of dimethyl sulfoxide (DMSO) solution of lPGS%-PTMC (10 mg/mL) was constantly added under stirring at 500 rpm. After set still for 1 h, the solution containing micelles self-assembled from lPGS%-PTMC was dialyzed extensively (MWCO = 3.5 kDa) against PB buffer for 24 h. Afterwards, the size, polydispersity and zeta potential of lPGS%-PTMC was measured by dynamic light scattering (DLS). The morphology of the formulated nano-assemblies was characterized by cryo-electron microscopy (cryo-EM).

**Propagation and purification of HSV-1 virus**

HSV-1-GFP was propagated in Vero E6 cells and titrated by plaque assays on Vero E6 cells. The virus was stored in -80 °C before usage.

For the virus purification, the HSV-1 virus from cell culture supernatant was firstly filtered through 0.45 μm filter and then centrifuged with 20% sucrose cushion for 2 h at 100, 000g at 4 °C. The pellet was resuspended in PBS and dialyzed against PBS for 24 h. Finally, the purified virus was fixed by 4% formaldehyde for 24h and stored at -80 °C.

**Inhibition of lPGS%-PTMC nano-assemblies against HSV-1 via plaque reduction assay**

2 × 10^4^ of Vero E6 cells were seeded in a 12-well plate to confluency one day prior to the assay. Then, each of the lPGS%-PTMC nano-assemblies varying sulfation ratio were serially diluted with DMEM medium by 10-fold and 100 μL of each dilution of each compound was incubated with 100 μL of virus solution containing green fluorescence encoded HSV-1 (approx. 2000 PFU/mL) for 45 min at 37 °C under 5% CO_2_. Afterward, 100 μL of the supernatant was aspirated for titration by plaque assay using Vero E6 cells and 500 μL of DMEM-Avicel as the overlay medium. The infected Vero E6 cells were fixed after 72 hours, and the images of plaques were processed with ImageJ, adjusting contrast, setting thresholds, analyzing particles with demanded particle size. The number of plaques was obtained as the number of particles. The inhibition ratio was calculated as the equation below:

$$\text{Inhibition} \% =\left( 1- \frac{\text{Number of plaques}}{\text{Number of plaques in virus control}} \right)\times100$$

**Virucidal assay**

For virucidal assay, the sample was diluted to 1 mg/ml and then incubated with HSV-1 solution (approx. 1×10^7^ PFU/mL) for 45min. Afterwards, the mixture with diluted by 10-fold and evaluated by plaque assay on VeroE6 cells.

**Fluorescence imaging of lPGS%-PTMC nano-assemblies inhibiting HSV-1**

The inhibition of lPGS%-PTMC nano-assemblies against HSV-1 was evaluated by both pre-infection assay and post-infection assay, and the infected cells in both cases were imaged by fluorescent microscopy. For the pre-infection assay, the Vero E6 cells were firstly seeded into a 24-well plate to confluency. Then each of the serially diluted of lPGS%-PTMC nano-assemblies (100 μL) were individually incubated with 100 μL of HSV-1 solution for 45 min at 37 °C under 5% CO_2_. Afterwards, to each well of Vero E6 cells, 200 μL of the compound pre-treated virus solution was added. The Vero E6 cells were incubated further for 24 hours. Then, the cell nuclei were stained by Hoechst and the Vero E6 cells were fixed by 4% formaldehyde. The infection of Vero E6 cells was imaged by ZEISS Axioscope 5 fluorescence microscope (ZEISS, Germany).

For the post-infection assay, Vero E6 cells were similarly grown in a 24-well plate to confluency before the infection. Then, to each well of Vero E6 cells, 100 μL of HSV-1 solution was added and incubated with the cells for 1 hour at 37 °C under 5% CO_2_. Afterwards, the cells were washed by PBS buffer and cultured with the lPGS%-PTMC nano-assemblies pre-mixed in DMEM medium for 24 hours. The cells were fixed, and the nuclei were stained by Hoechst for fluorescence microscopy as mentioned above.

**Cell viability assay of lPGS%-PTMC nano-assemblies**

The cellular biocompatibility of lPGS%-PTMC nano-assemblies varying from sulfation ratio was investigated with Vero E6 cells. Generally, the Vero E6 cells were seeded into a 96-well plate at the density of 5 × 10^3^ cells per well and incubated at 37 °C overnight. Then 10 µL of lPGS%-PTMC nano-assemblies of different sulfation rate and their serially diluted solution was separately added to different wells and incubated further for 24 hours. The well added with 10 µL of PBS buffer was considered as the control group. Afterwards, 10 µL of the cell counting kit-8 solution was added to each well. After one-hour incubation, the absorbance of each well at 450 nm was determined by microplate reader and the relative cell viability was calculated by comparing with the control group.

**Fluorescence imaging of lPGS%-PTMC nano-assemblies inhibiting Omicron BA.5.**

Briefly, Vero E6 cells were seeded in 96-well plates. After being washed with PBS and refilled with 90µL of infection medium (MEM, 1% FBS, P/S), 10 µL of samples were added in the medium and incubated on the cells for 90 min. Finally, 10 µL of virus dilution (MOI = 0.1) was added to each well and the plates were incubated at 37 ^o^C for 45 min. Subsequently, medium was removed and cells were washed once with PBS. Infection medium was replenished to 100 µL and cells were incubated at 37 °C for 48 h. After 48 h, cells were washed and fixed with 4% PBS-buffered formaldehyde. Later, the cells were permeabilized by 0.25% Trixton X-100 and labelled by SARS-CoV-2 nucleocapsid (N) Protein Antibody and Alexa 488 conjugated goat anti-mouse IgG. The cell nuclei were stained by Hoechst for fluorescence microscopy.


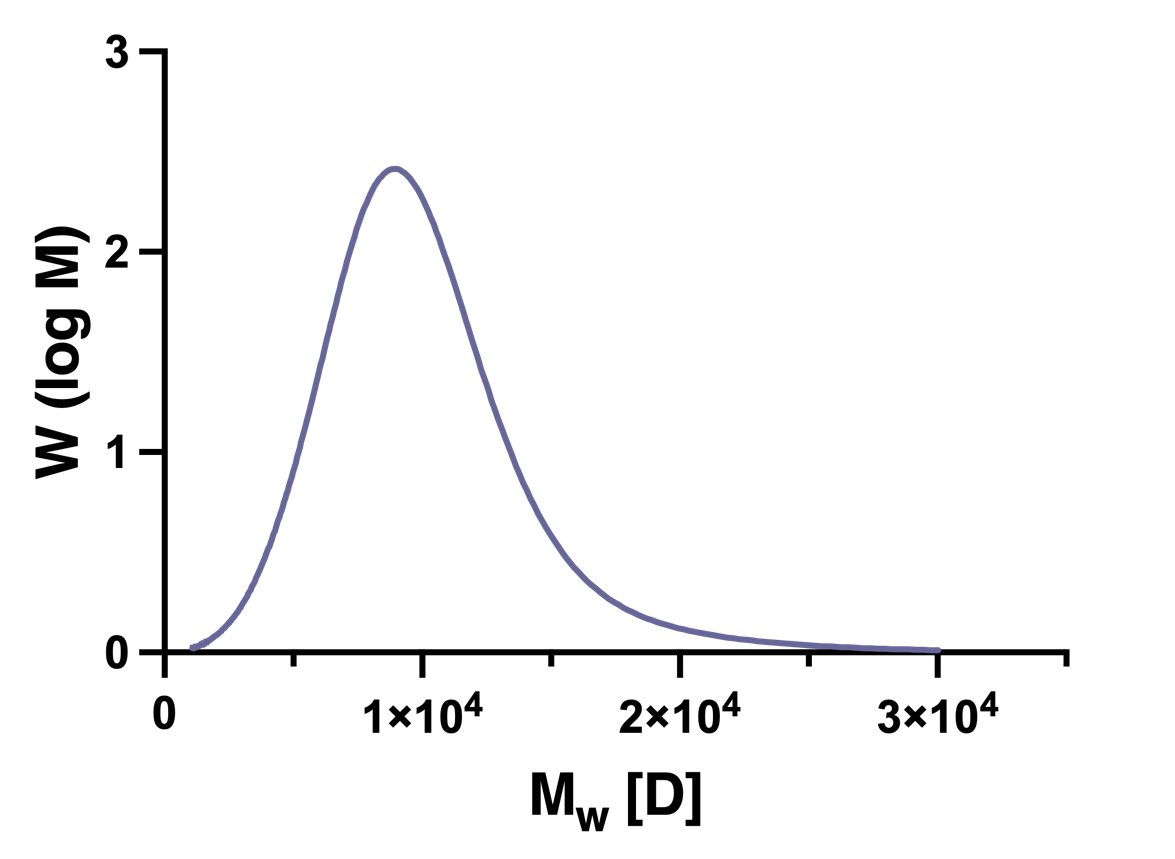

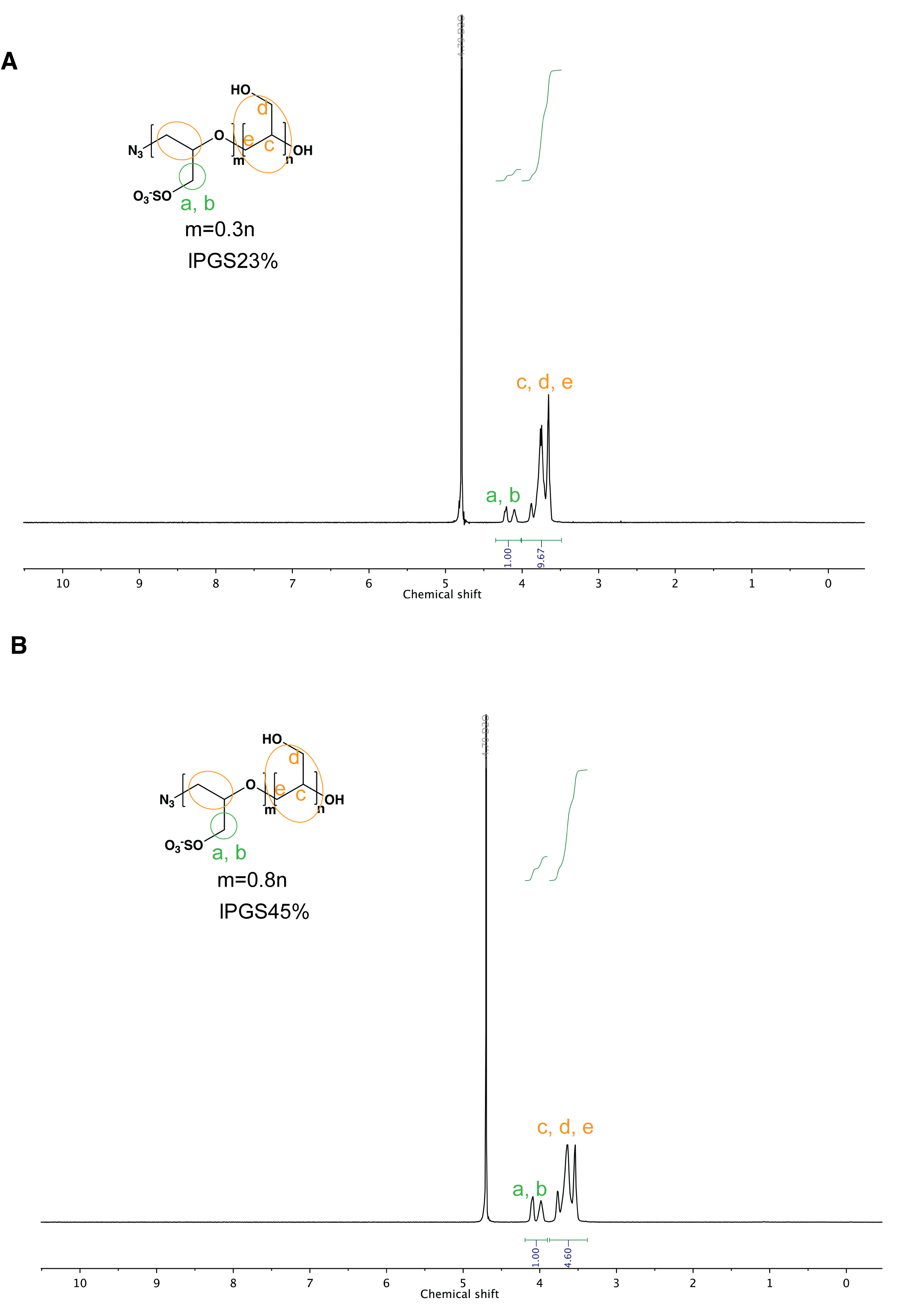


**Figure S1.** Molecular weight of linear polyglycerol measured by gel permeation chromatography.


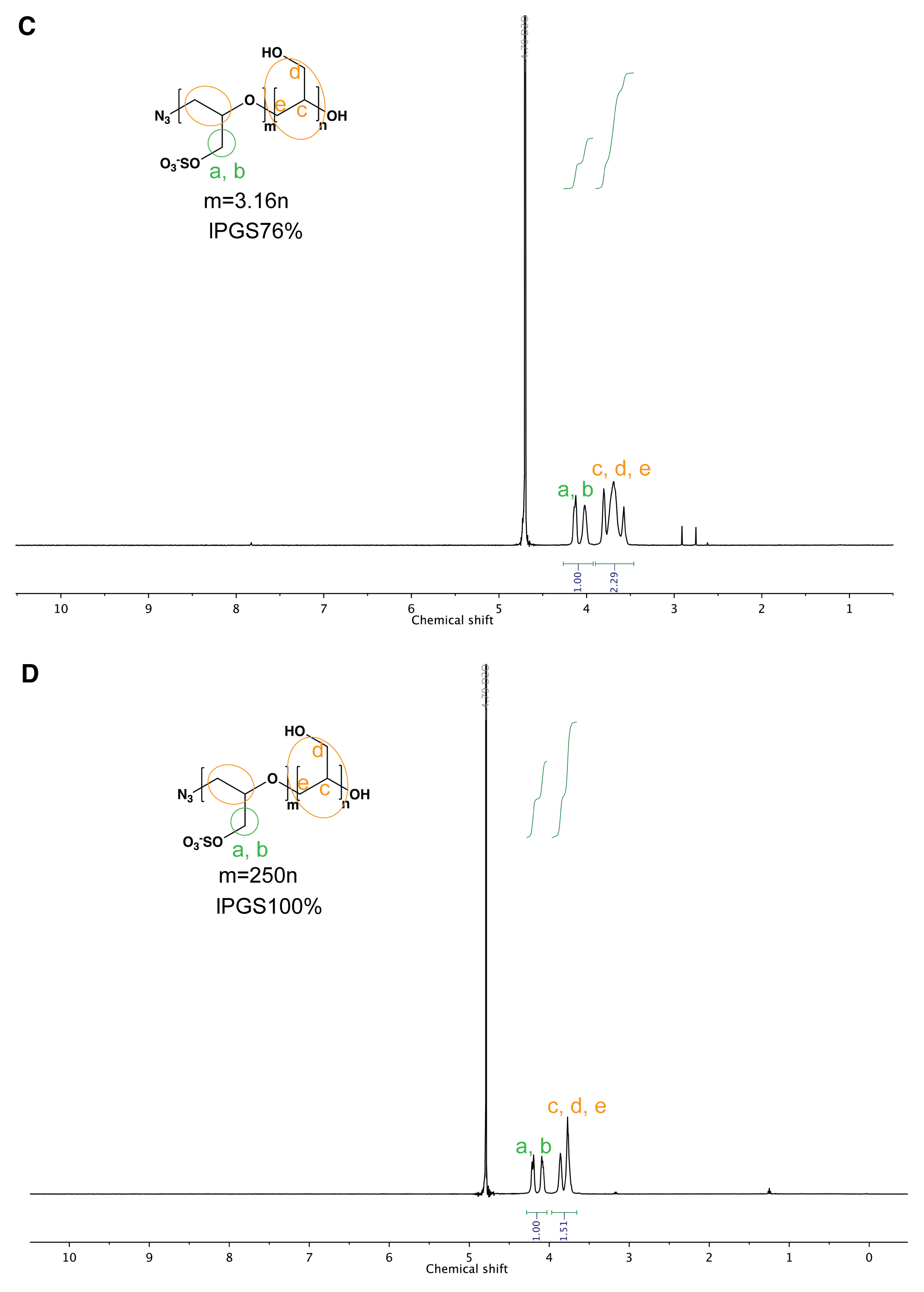


**Figure S2.** ^1^H-NMR spectrum of (A) 23% sulfated linear polyglycerol, (B) 45% sulfated linear polyglycerol, (C) 76% sulfated linear polyglycerol and (D) 100% sulfated linear polyglycerol.


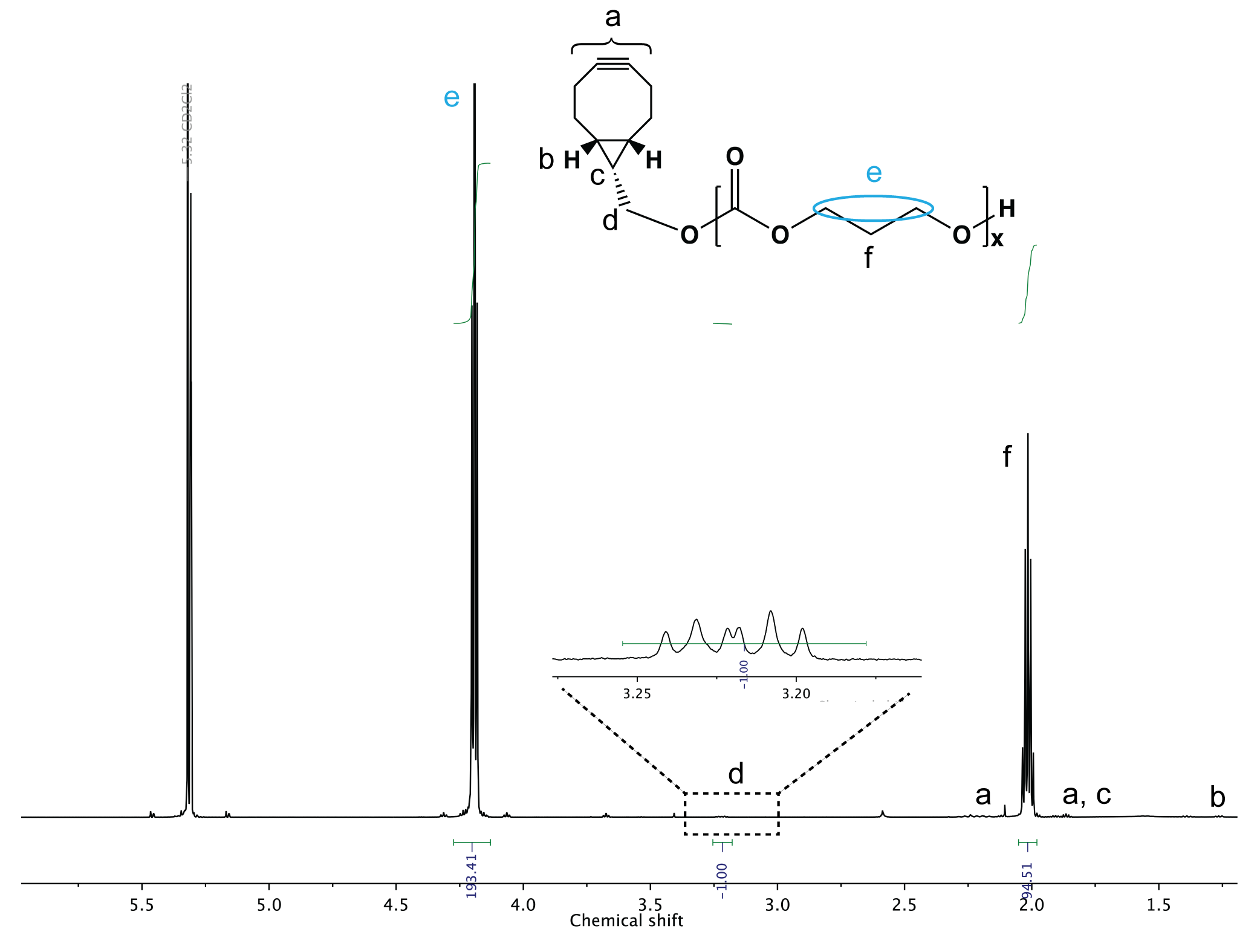


**Figure S3.** ^1^H-NMR spectrum of BCN functionalized polytrimethylene carbonate.


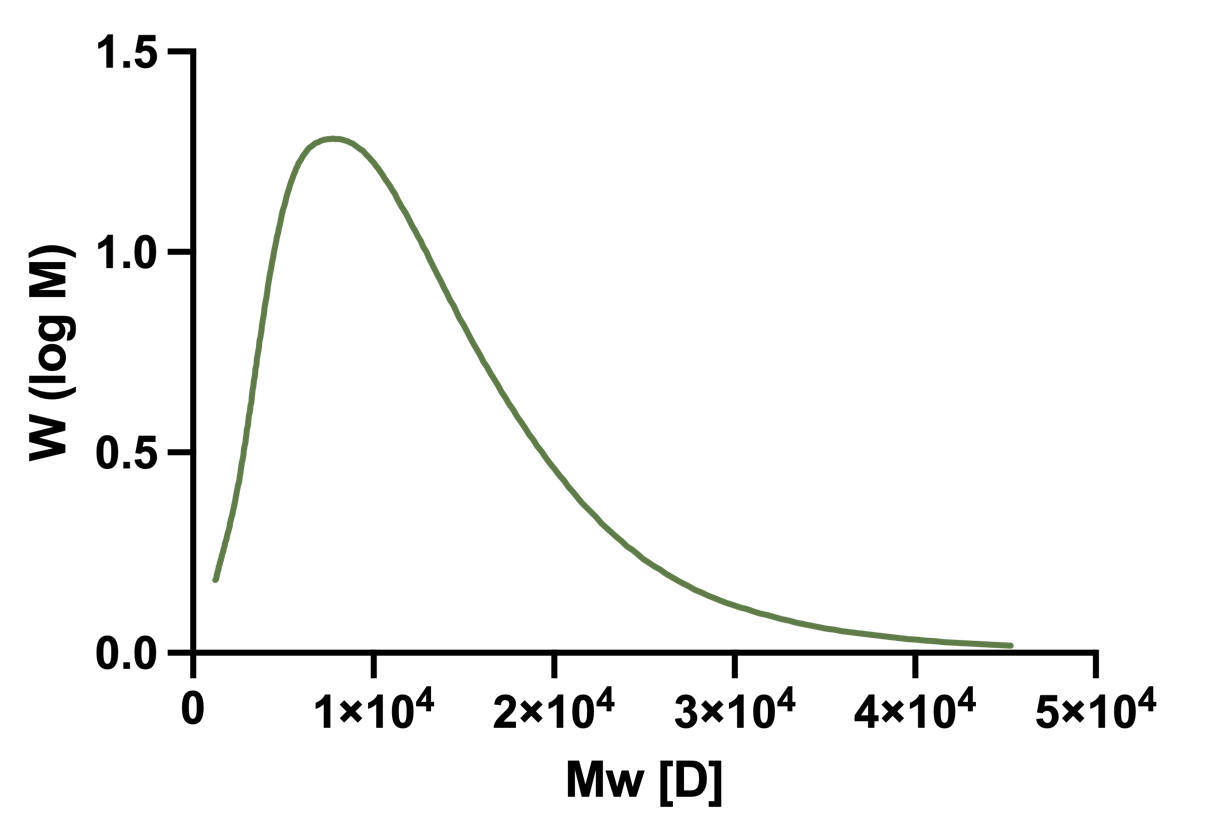


**Figure S4.** Molecular weight of BCN functionalized polytrimethylene carbonate measured by gel permeation chromatography.


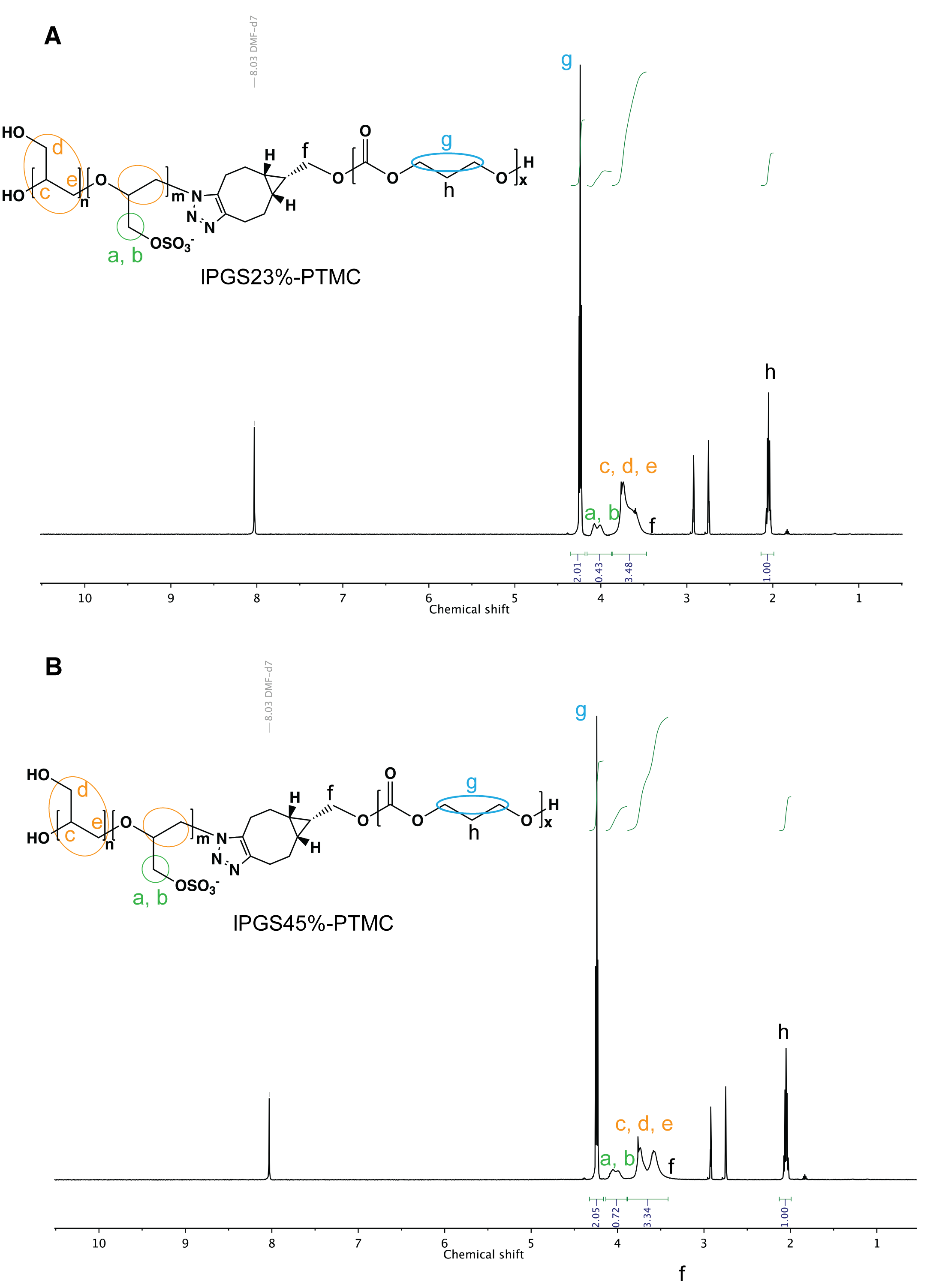


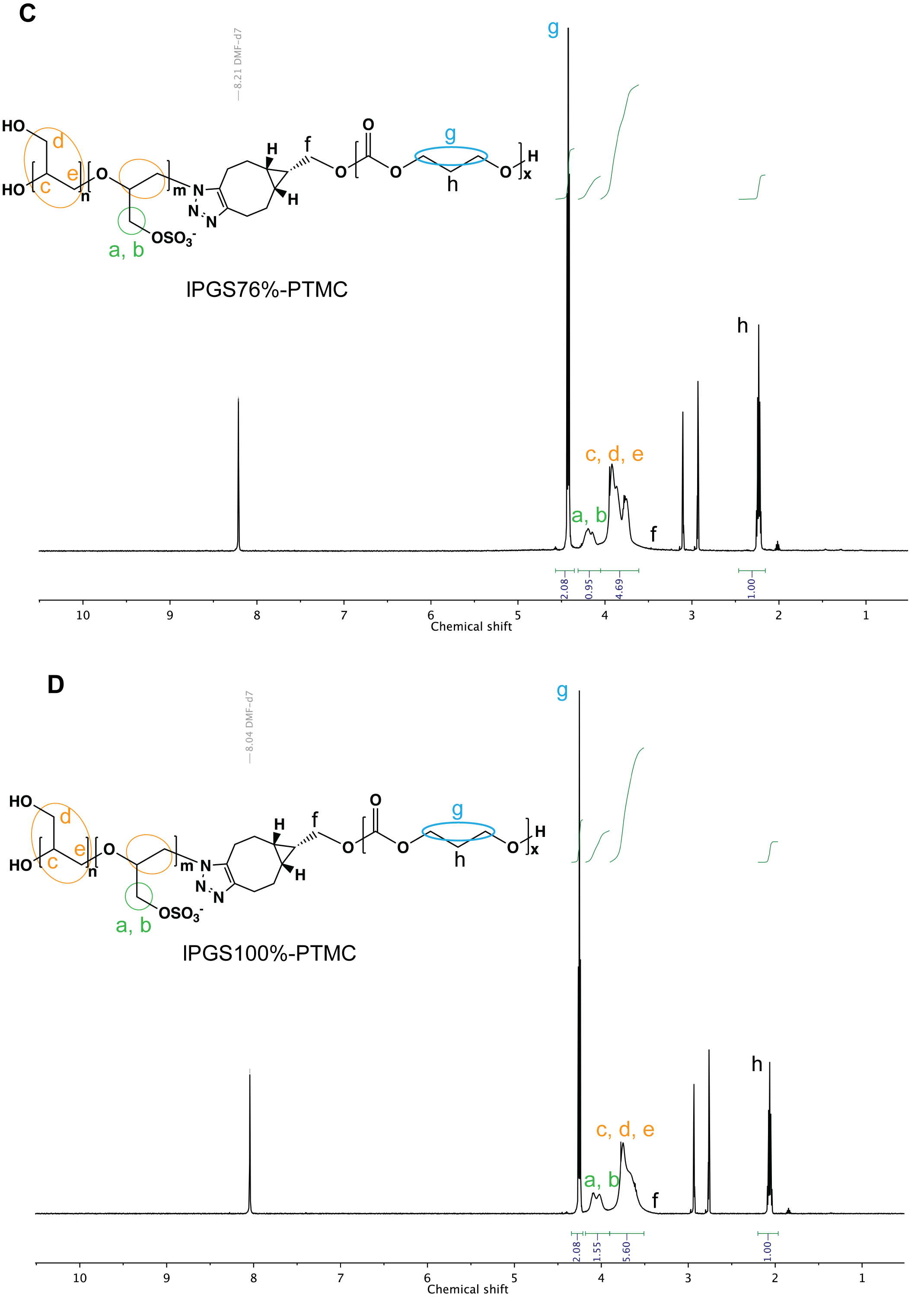


**Figure S5.** ^1^H-NMR spectrum of (A) lPGS-23%-PTMC, (B) lPGS-45%-PTMC, (C) lPGS-76%-PTMC l and (D) lPGS-100%-PTMC.


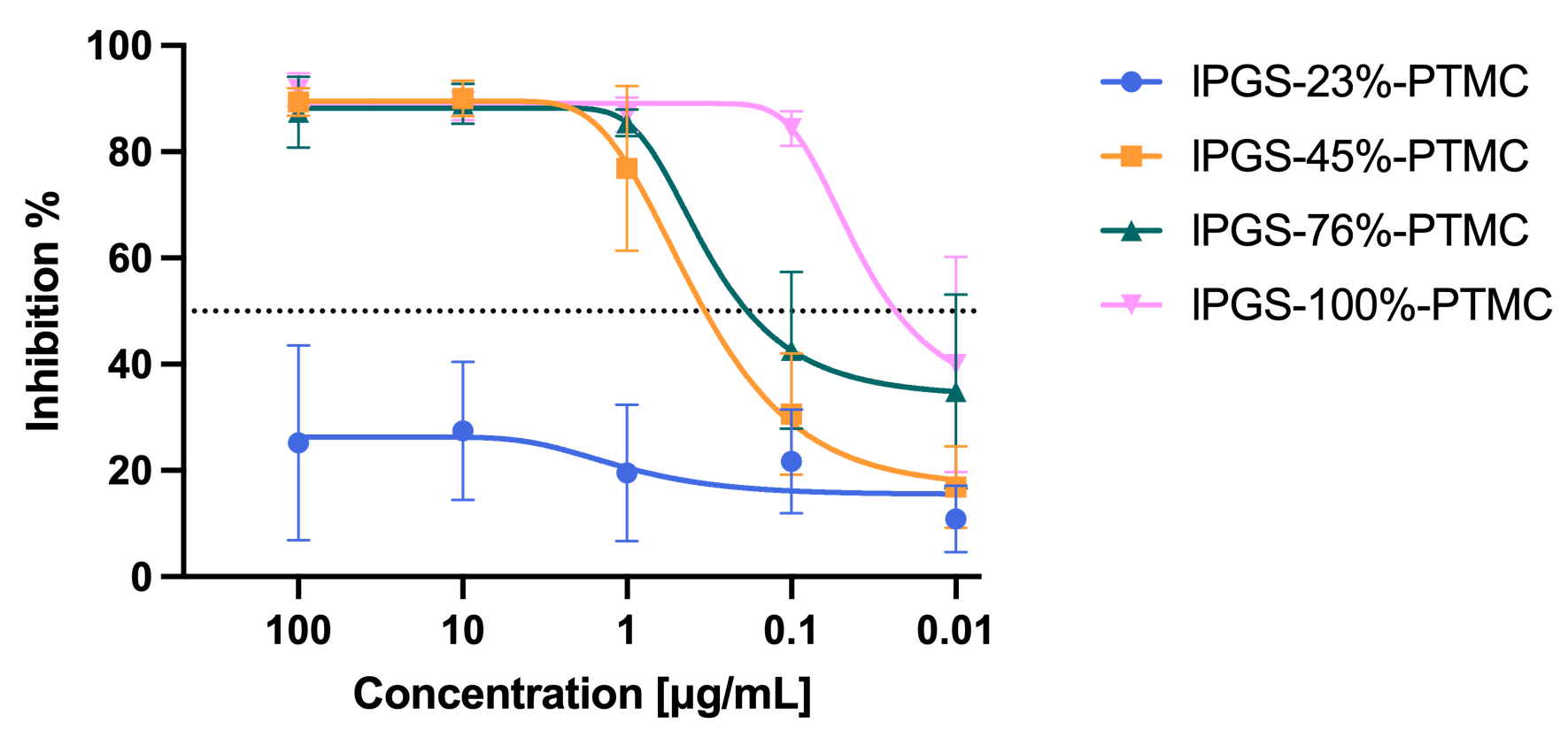

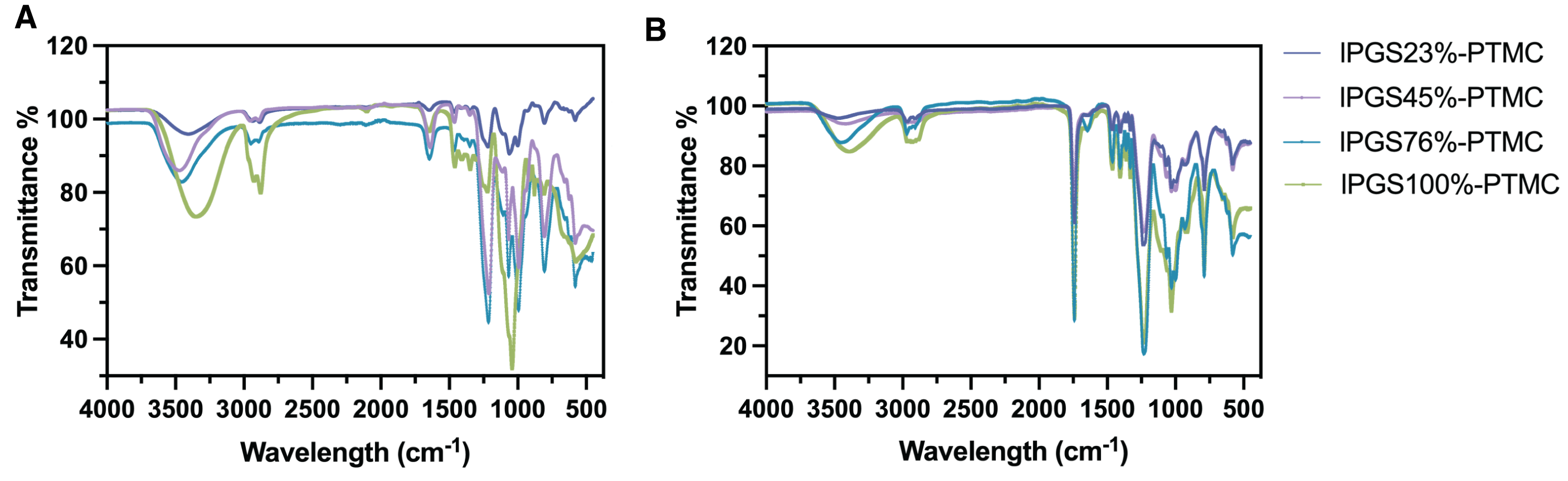


**Figure S6.** FTIR spectrum of (A) lPGS% and (B) lPGS%-PTMC varying sulfation ratio.

**Figure S7.** Quantification of inhibition efficiency from plaque reduction assay (mean ± SD, n = 5, analyzed by log(inhibitor) vs. response – variable slope model embedded in Prism).


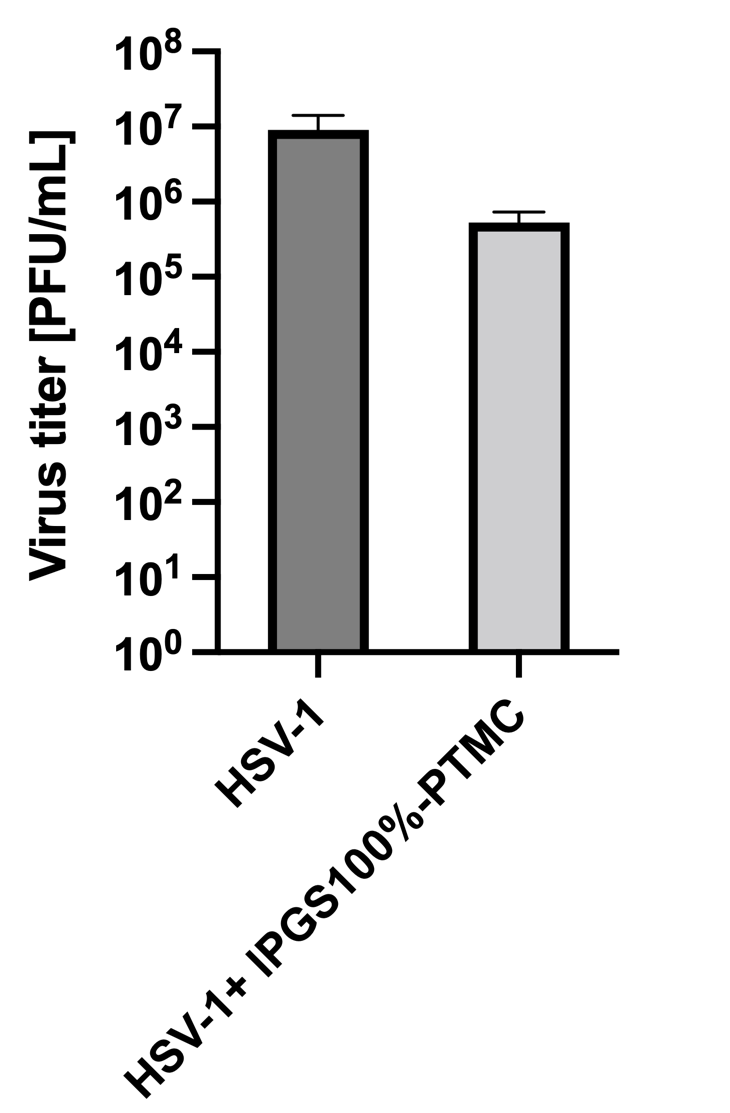


**Figure S8.** Quantification of virucidal efficiency of lPGS100%-PTMC nano-assemblies (mean ± SD, n = 6).


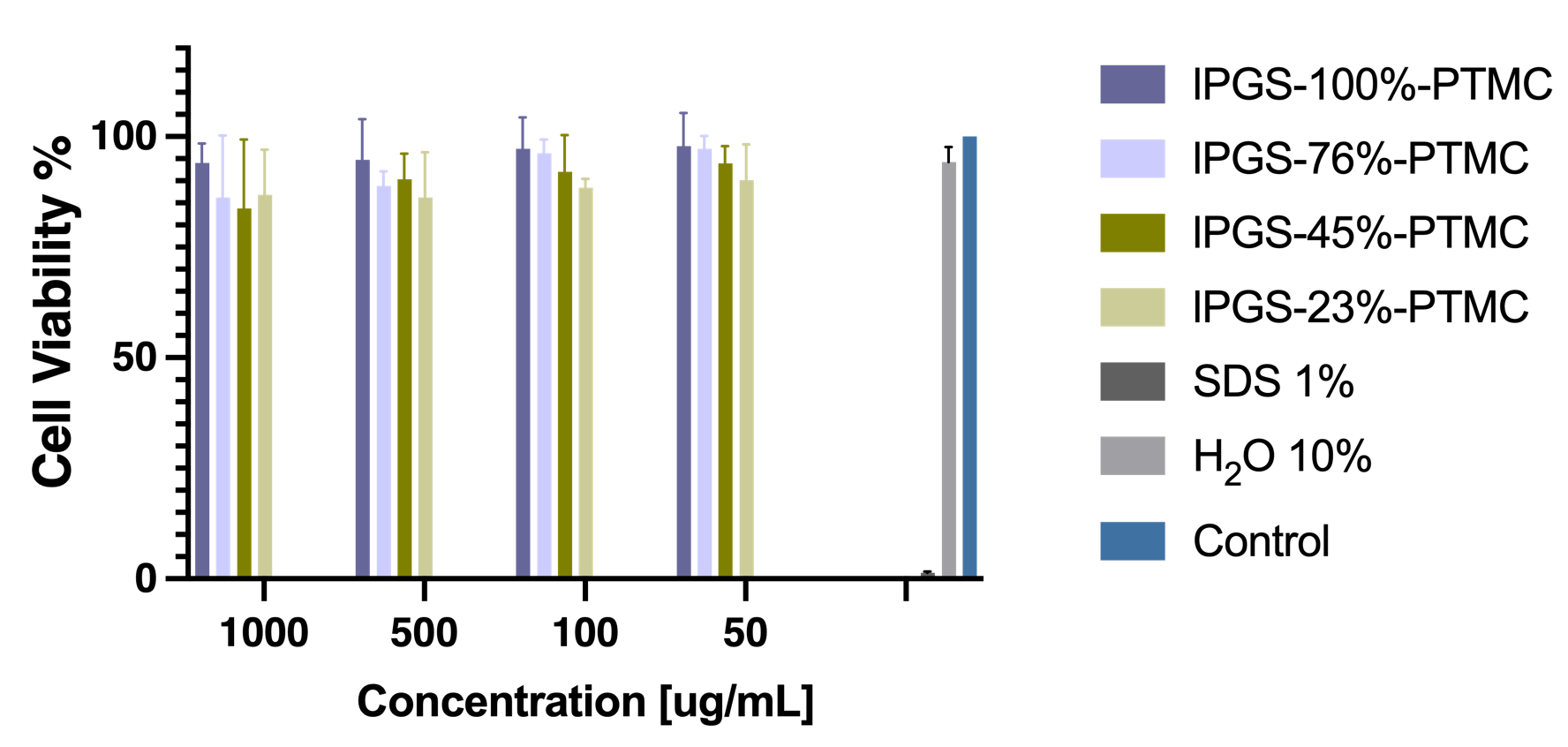

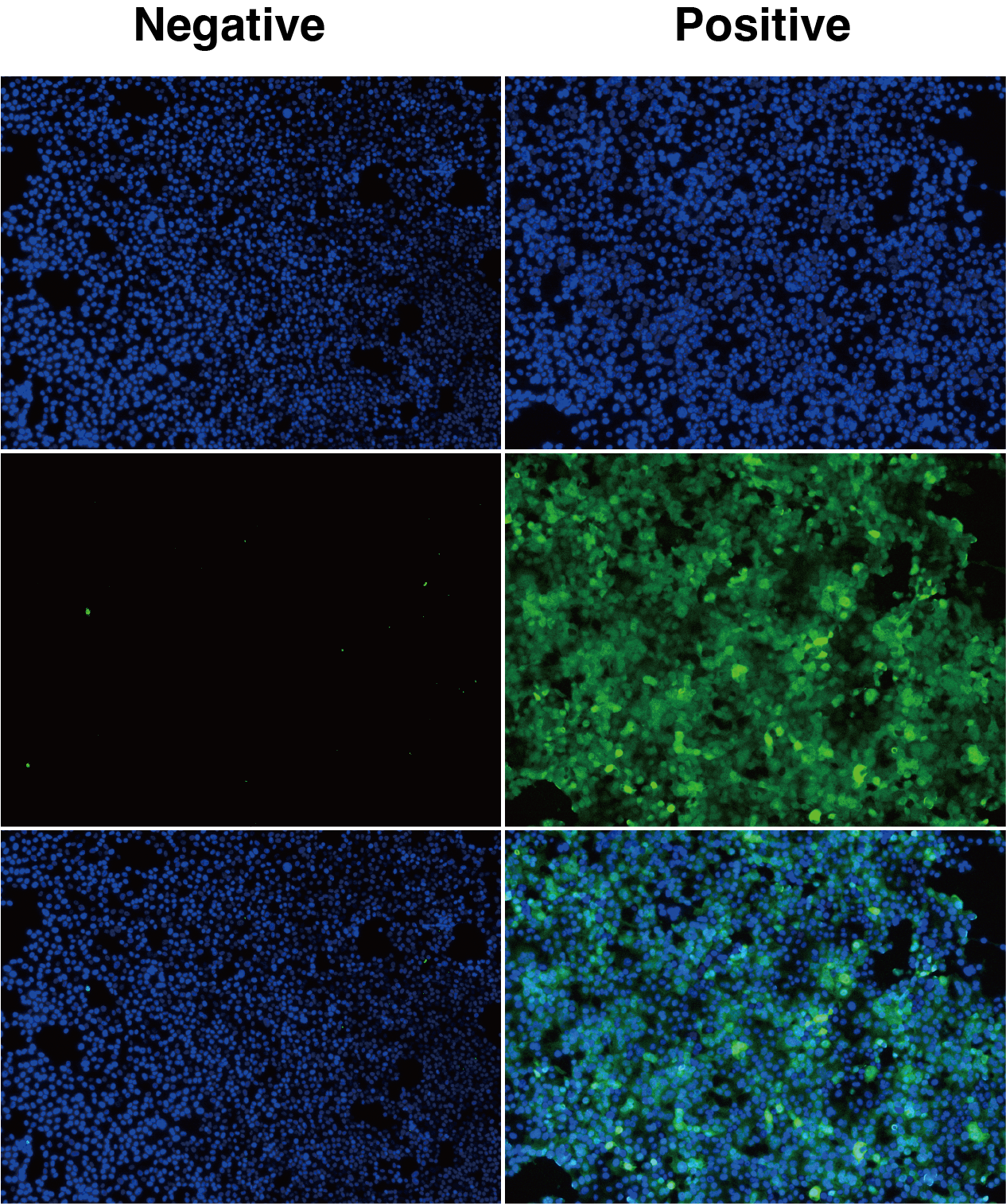


**Figure S10.** CCK-8 assay of Vero E6 cells treated by lPGS%-PTMC nano-assemblies with varying sulfation ratios.

**Figure S9.** Negative (uninfected Vero E6 cells) and positive (infected Vero E6 cells without treatment) control groups in the pre- and post-infection assay tested on Omicron BA.5.

**References**

[1] M. Tully, N. Hauptstein, K. Licha, L. Meinel, T. Lühmann, R. Haag, *Journal of Pharmaceutical Sciences* **2022**, *111*, 1642-1651.

[2] J. Wei, H. Meng, B. Guo, Z. Zhong, F. Meng, *Biomacromolecules* **2018**, *19*, 2294-2301.

[3] H. Türk, R. Haag, S. Alban, *Bioconjugate chemistry* **2004**, *15*, 162-167.
